# Bronchoscopy-guided intervention therapy with extracorporeal membrane oxygenation support for advanced cancer metastasis to the central airway: A case report

**DOI:** 10.1097/MD.0000000000019488

**Published:** 2020-03-13

**Authors:** Wei Yu, Pengcheng Zhou, Keling Chen, Wenjun Tang, Qianming Xia, Junmei Ma

**Affiliations:** Department of Respiratory Medicine, Hospital of Chengdu University of Traditional Chinese Medicine, Chengdu, Sichuan province, P.R. China.

**Keywords:** bronchoscopy, extracorporeal membrane oxygenation, interventional therapy, malignant tumor, severe airway stenosis

## Abstract

**Introduction:**

Dyspnea due to tracheal invasion by malignant tumors is a common oncological emergency that is difficult to manage, and a common cause of death among patients with advanced cancer. Bronchoscopy-guided intervention therapy under conventional ventilation is very risky for patients with severe central airway stenosis. Extracorporeal membrane oxygenation (ECMO) provides strong cardiopulmonary support, but is rarely used in bronchoscopy-guided interventional therapy.

**Patient concerns:**

The patient had advanced esophageal cancer with metastases to the trachea and left and right main bronchi. Despite several sessions of radiotherapy, chemotherapy, and bronchoscopy-guided intervention therapy, the tumor in the airway became enlarged, the lumen was severely narrow, and the patient experienced respiratory distress.

**Diagnosis:**

A thoracic computed tomography scan performed at our hospital revealed invasion of the trachea and opening of the left and right main bronchi by the esophageal cancer, blockage of the stent by the tumor, and severe luminal narrowing. An emergency bronchoscopy showed slit-like stenosis of the middle and lower part of the trachea and the left and right main bronchi, and the tumor was highly vascular.

**Interventions:**

To reduce the risk of major airway bleeding and asphyxia during bronchoscopy under conventional ventilation, we finally performed argon plasma coagulation with a high frequency electric knife and cryotherapy with ECMO support.

**Outcomes:**

We successfully cleared the tumor tissue in the airway under ECMO support. The trachea and left and right main bronchi recovered smoothly, and the patient was soon discharged.

**Conclusion:**

ECMO can meet the oxygenation needs during bronchoscopy-guided intervention therapy. For patients with severe central airway obstruction due to malignant tumors, ECMO should be considered if conventional respiratory support cannot guarantee the safety of surgery.

## 1 Introduction

Advanced central airway metastasis of malignant tumors is very difficult to manage clinically. Due to cachexia after surgery, chemoradiotherapy, or molecular targeted therapy, these conventional treatments are difficult to reapply to such patients.^[1]^ Bronchoscopic intervention is safe, minimally invasive, and well tolerated, and is increasingly used in various benign and malignant conditions characterized by central airway stenosis.^[2,3]^ Traditional respiratory support methods, including nasal oxygen inhalation, respiratory mask, implanted hard endoscopy, laryngeal mask or tracheal intubation, and mechanical ventilation, are only suitable for patients with mild airway stenosis, and have general health status. Among patients with severe airway stenosis, tumors are highly vascular, and prone to major bleeding during interventional therapy especially in patients with cardiopulmonary failure, conventional respiratory support is riskier and it is difficult to meet the needs of bronchoscopy-guided interventional therapy.^[4]^ In recent years, extracorporeal membrane oxygenation (ECMO) technology has developed, and has been widely used in acute respiratory distress syndrome, cardiopulmonary failure, and various complications of cardiac and thoracic surgery.^[5,6]^ Although ECMO provides adequate cardiopulmonary support function, it is rarely used in bronchoscopy-guided interventional therapy for advanced tumors.

We herein report a case of esophageal cancer metastasis to the central airway. Although the patient had undergone multiple interventional therapies at other hospitals and had received implantation of a tracheal stent, the tumor progressed rapidly, completely occluding the trachea and the left and right main bronchi. Because the tumor was highly vascular, the airway was severely narrowed, and the interventional treatment increased the risk of suffocation due to massive bleeding. Although the tracheal intubation was also very risky, we finally decided to perform high-frequency electrosurgical argon plasma coagulation and cryotherapy with ECMO support. The patient was soon discharged from the hospital.

## 2 Case presentation

**Figure 1:**
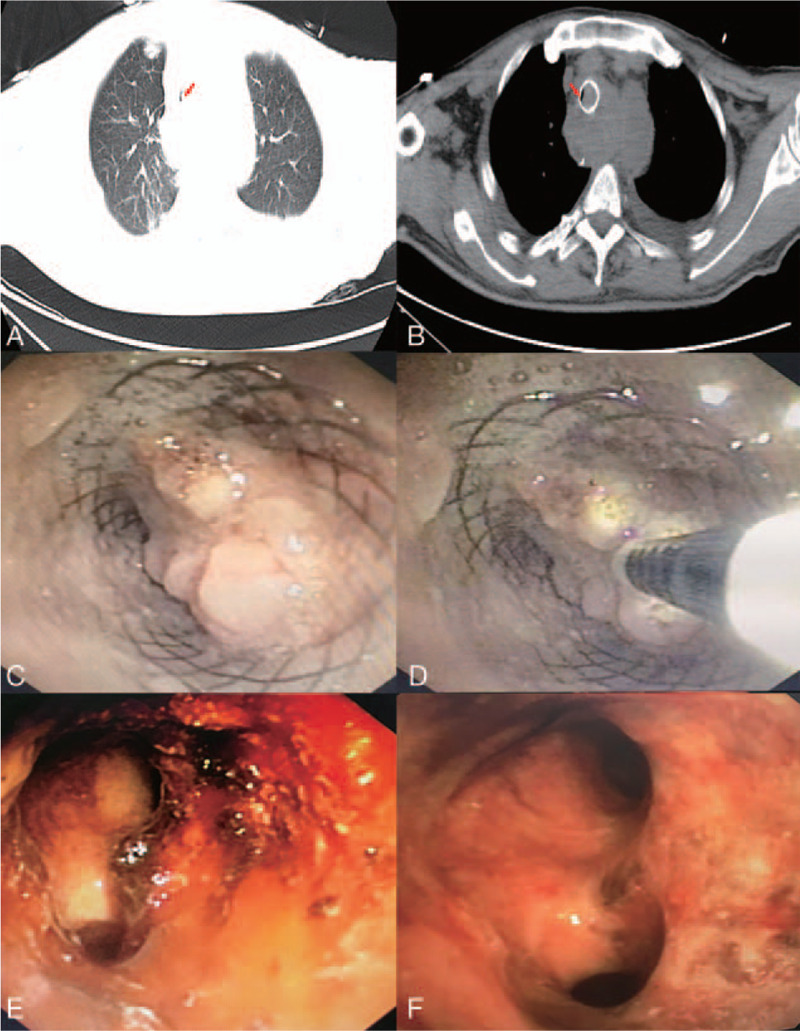
(A and B) Chest computed tomography scan showing tracheal invasion by the esophageal tumor, as well as invasion of the opening of the left and right main bronchi; the tumor tissue blocked the stent and the lumen was severely narrowed (red arrow). (C) Bronchoscopy showing the middle and lower parts of the trachea and slit-like stenosis of the left and right main bronchi, and the highly vascularized tumor. (D) Argon plasma coagulation was used during bronchoscopy-guided intervention therapy. (E and F) The trachea and the left and right main bronchi were recanalized after therapy.

The patient was a 58-year-old farmer who was diagnosed with esophageal squamous cell carcinoma a year ago. Subsequently, surgery, multiple radiotherapy, and chemotherapy were performed at a local hospital. Tracheal invasion by the tumor was discovered due to dyspnea 3 months ago; subsequently, argon plasma coagulation, carbon dioxide cryoablation, and tracheal stent placement were performed at a university hospital. The patient developed severe dyspnea again 3 days before presentation. A chest computed tomography (CT) scan and bronchoscopy performed at a county hospital revealed that the trachea was almost completely occluded by the tumor. Considering that the risk of interventional therapy was too high, the patient was referred to our hospital for further treatment. Physical examination at our hospital showed that the patient was in respiratory distress; the respiratory rate was 40 cycles/min, the blood pressure was 137/87 mmHg, and the peripheral capillary oxygen saturation (SpO2) could only be maintained at 80% with the support of a noninvasive ventilator. In addition, the patient had reduced breath sounds and one could hear wet rales. A chest CT scan showed that the esophageal tumor had invaded the trachea and the opening of the left and right main bronchi, the tumor had blocked the stent, and the lumen was severely narrow (Fig. 1A and B). There was bronchiectasis and infection of the right lung. Except for an elevated white blood cell count, there were no obvious abnormalities on biochemistry and coagulation, and an electrocardiogram yielded normal findings. An emergency bronchoscopy showed slit-like stenosis of the middle and lower parts of the trachea and the left and right main bronchus, and the tumor was highly vascular (Fig. 1C). To avoid the risk of major airway bleeding and asphyxia during bronchoscopy under conventional ventilation, we finally decided to perform bronchoscopy-guided interventional therapy with ECMO support (V-V ECMO method), using a heparin-coated membrane lung.

We first inserted the F22 cannula into the left femoral vein of the patient, and the F16 venous cannula into the right internal jugular vein percutaneously. The direction of the pipe connection was as follows: left femoral vein—centrifugal pump—membrane lung - right internal jugular vein. The circulatory system was pre-filled with Wanwen injection 1000 mL, and continuously infused with heparin during ECMO. The mean arterial pressure, SpO2, hematocrit, and activated clotting time (ACT) during transfusion were monitored. The ECMO speed was 3000 rpm, the blood flow velocity was 3 L/min, the average arterial pressure was maintained at (90 ± 10) mmHg, and the ACT was maintained at 250 seconds. Considering the difficulty and risk of surgery, we decided to perform bronchoscopy-guided intervention therapy twice.

The intervention therapy method used is described in Figure 1D. The high-frequency electric knife was used to cut the tumor tissue, argon plasma coagulation was used to achieve hemostasis, and cryotherapy was used to destroy tumor cells followed by ablation. The trachea and left and right main bronchi were mostly recanalized after the first therapy (Fig. 1E). The amount of blood lost intraoperatively was about 50 mL, the SpO2 was maintained at 90% to 95%, and the remaining vital signs were stable. Postoperatively, we gradually stopped ECMO support; simultaneously, mechanical ventilation was administered. The patient suddenly experienced worsened dyspnea 12 hours after surgery and the ventilator monitor showed increased airway resistance. An emergency bronchoscopy showed that the left and right main bronchi were completely blocked by the blood clot. The blood clot in the patient's airway was considered to be caused by systemic heparinization during ECMO. The dyspnea significantly improved after clearing the blood clots and administering protamine to neutralize heparin. On the second day, we completely removed the remaining tumor tissue in the patient's airway under mechanical ventilation, with little intraoperative bleeding (Fig. 1F). On the third day, the patient was discharged after successful extubation.

## 3 Discussion

Dyspnea is a common manifestation of oncologic emergencies caused by malignant tumor invasion or compression of the tracheobronchial tree. It is also a common cause of death among patients with advanced cancer.^[1]^ The traditional treatment method is not ideal in time and effect, and even directly leads to the death of some patients.^[2]^ Patients can only be saved by quickly releasing the airway obstruction and opening the respiratory passage. In recent years, endoscopic interventional therapy has become the first choice for timely and effective treatment of airway stenosis caused by various tumors.^[3]^ For intraluminal lesions that previously required surgery or could not be treated at all, bronchoscopy-guided interventional therapy can be used to obtain better results.^[4]^ However, severe obstruction of the central airway caused by some tumors is still a thorny problem during endoscopic surgery. Conventional respiratory support cannot solve the problem of intraoperative hypoxia. For example, a wide range of tumor lesions cannot be managed by tracheostomy because the tumor is highly vascular. Interventional treatment increases the risk of major bleeding or severe bilateral obstruction of the main bronchus; even if administered, mechanical ventilation cannot solve the issue of lack of oxygen. These patients are advanced cancer patients and have lost the opportunity for radical surgery. Therefore, there is currently a search for a new method to solve the problem of oxygen supply in patients undergoing endoscopic surgery.

ECMO, also known as extracorporeal life support, is an effective adjuvant therapy for life support based on the extracorporeal circulation system and using extracorporeal circulation technology. Its main purpose is to provide blood oxygenation and remove carbon dioxide, to ensure effective blood supply to the body; by providing emergency and critically ill patients with respiratory and circulatory support, it plays an important role in emergency and critical care.^[5]^ In the in vitro life support application guidelines developed by the Extracorporeal Life Support Organization in 2009, it was noted that for patients with severe acute cardiopulmonary failure, after providing the best conventional treatment, ECMO should be used. Using ECMO, the mortality rate reduces from 80% to 50%.^[6]^ The treatment modes of ECMO mainly include venous-arterial ECMO (V-A ECMO) and venous-venous ECMO (V-V ECMO). The former can partially or completely replace cardiopulmonary function, and the latter is mainly used for simple respiratory insufficiency or exhaustion. In respiratory failure, the most common indications are acute respiratory distress syndrome, pneumonia, trauma, or lung transplant failure.^[7,8]^ In heart failure mainly due to acute myocardial infarction and cardiogenic shock due to arrhythmia, life support may be required before heart transplantation.^[9]^ With the development of technology, the use of ECMO has become more and more extensive. Krecmerova et al^[10]^ successfully performed massive alveolar lavage with severe alveolar proteinosis under the support of ECMO. Pitcher et al^[11]^ successfully performed emergency surgery on patients with massive hemoptysis with ECMO support. Natt et al^[12]^ successfully performed balloon dilatation and tracheal stenting with V-V ECMO support in patients with severe tracheal occlusion after tracheal intubation, and the patient's postoperative dyspnea was significantly restored. In addition, ECMO is also widely used in noncardiopulmonary system diseases. Kang et al^[13]^ used ECMO to provide the necessary life support to patients with advanced hematological tumors. Fan et al^[14]^ used ECMO to provide life support to donor organs in brain-dead patients and increased organ donation rates.

Although ECMO is widely used in conditions such as cardiopulmonary failure and shows provides respiratory and circulatory support function, there are few reports of interventional procedures for airway metastasis of advanced malignant tumors, and their safety and complications are unclear. Acute and progressive dyspnea is the main symptom of patients with malignant tumors with central airway obstruction. When the airway is severely occluded, compressed, infiltrated, or even bleeds, the patient experiences worsened respiratory distress, suffocation, or even death. The treatment is very difficult. For patients with traditional radiotherapy and chemotherapy, the curative effect is limited, and the bronchoscopic intervention under conventional respiratory support is also extremely risky. Any attempt at intubation and hemorrhage during tumor ablation can result in complete airway occlusion and death. Therefore, for patients with severe airway obstruction due to malignant tumors, the key to successful bronchoscopy-guided interventional therapy is the improvement of gas exchange and maintenance of the necessary oxygen synthesis. Due to its ability to replace cardiopulmonary function in whole or in part, ECMO has become increasingly important in recent years in the treatment of advanced tumor airway obstruction via bronchoscopy.

Hong et al^[15]^ reported that 15 patients with malignant tumors experienced severe airway obstruction with successful interventional treatment with ECMO support; 8 patients were treated with rigid bronchoscopy and 7 patients with airway stents. Using ECMO, Kim et al^[16]^ successfully performed bronchoscopic tumor resection in an 88-year-old patient with tracheal metastases of a mediastinal teratoma. The operation was safely completed and there were no complications. Yi et al^[17]^ reported the cases of 2 patients with primary bronchial tumors who underwent endoscopic tumor resection with ECMO support and received airway stent placement. Our patient experienced central airway invasion secondary to esophageal cancer. Although the patient had undergone multiple interventional ablation and tracheal stent placement at another hospital, the tumor progressed rapidly, completely blocking the trachea and the left and right main bronchi. The tumor had a rich blood supply, and the interventional treatment could very easy cause suffocation due to massive hemorrhage. Although tracheal intubation was also very risky, we finally decided to perform bronchoscopy-guided interventional therapy with ECMO support. We successfully cleared the airway of tumor tissue eventually. Our case showed that ECMO had great value in bronchoscopy-guided intervention therapy.

Although ECMO provides adequate cardiopulmonary support, it also has complications such as hemorrhage, hemolysis, embolism, infection, and edema.^[18]^ Sy et al^[19]^ systematically evaluated the complications of approximately 1496 patients in 26 studies treated with ECMO. The results showed that bleeding was the most common complication of ECMO, with a prevalence of 27%. Major bleeding requiring reoperation was the most common bleeding event. The overall prevalence of thromboembolic events was 8%, with limb ischemia, intravascular coagulation, and stroke being the most frequently reported events. The overall prevalence of in-hospital mortality was 59%. Hong et al^[16]^ reported the case of a patient with malignant tumor who experienced major airway bleeding after tumor resection with ECMO support, and eventually died. Our patient experienced successful relief of tracheal tumor obstruction for the first time with ECMO support. The vital signs recovered quickly after the operation, but the patient soon experienced respiratory distress again. Emergency bronchoscopy revealed a large amount of blood clots blocking the airway. Considering the amount of heparin used during ECMO, the patient's dyspnea was rapidly relieved by reducing the amount of heparin, administering protamine to antagonize heparin, closely monitoring blood coagulation, and clearing airway clots. The patient did not experience oozing of blood in the airway again during the second intervention. It has been reported that using extracorporeal loop surface heparin coating technology to avoid non-urgent invasive procedures and monitoring ACT can effectively reduce the incidence of bleeding.^[20]^ Hemorrhage commonly occurs during the clinical ablation of airway tumors, and systemic heparinization during ECMO is bound to further increase the risk of coagulopathy such as major bleeding and embolism. Therefore, further research on the amount and timing of heparin should be conducted in the future to improve the safety of interventional surgery.

In addition, for airway metastasis of malignant tumors, some scholars believe that all surgical operations should be completed under cardiopulmonary bypass (CPB). Fan et al^[21]^ proposed that for patients with larger tumors occupying >70% of the lumen, temporary extracorporeal circulation must be used for surgical treatment. Gao et al^[22]^ posit that extracorporeal circulation (CPB) should be used for the surgical treatment of tracheal tumors. However, CPB is more likely to increase postoperative bleeding and potential complications such as liver and kidney dysfunction, and the establishment of CPB technology is difficult and time-consuming, causing great trauma to patients and more complications in later infections, so it is not an ideal support method.^[23]^ ECMO can overcome the above shortcomings of CPB, but is relatively expensive, making it difficult to achieve clinical development. The advantages of ECMO in bronchoscopy-guided interventional treatment of malignant tracheal tumors are as follows: even though the intervention and anesthesia share the airway, ECMO eliminates the interference of tracheal intubation, making the surgical field more open and clear; it allows the surgeon more operation time and room to perform the surgery in an orderly manner; it maintains stable oxygenation and hemodynamics during surgery; and it results in fewer complications such as bleeding compared to extracorporeal circulation. From the experience of the diagnosis and treatment of our patient and the previous literature, for the severe stenosis caused by the metastasis of malignant tumor to the central airway, it can be considered to complete the bronchoscopy intervention of the tumor with ECMO support, if in the routine respiratory support methods cannot meet the safety of the operation. In-depth clinical research on the safety and complications of ECMO is needed in the future.

## 4 Conclusions

Malignant tumor airway metastasis is an oncologic emergency and is characterized by a very high mortality rate. For patients with severe airway obstruction caused by malignant tumors, ECMO should be considered if conventional respiratory support cannot guarantee the safety of bronchoscopy.

## Author contributions

Conception and design: Pengcheng Zhou

Administrative support: Junmei Ma and Qianming Xia

Provision of study materials or patients: Wenjun Tang

Collection and assembly of data: Wei Yu

Data analysis and interpretation: Keling Chen,

Manuscript writing: All authors

(VII)Final approval of manuscript: All authors

**Conceptualization:** Pengcheng Zhou.

**Data curation:** Wei Yu.

**Formal analysis:** keling Chen.

**Methodology:** Wenjun Tang, Junmei Ma.

**Project administration:** Qianming Xia, Junmei Ma.

**Resources:** keling Chen.

**Supervision:** Qianming Xia.

**Writing – original draft:** Wei Yu, Pengcheng Zhou, keling Chen, Wenjun Tang.

**Writing – review & editing:** Wei Yu.

Pengcheng Zhou orcid: 0000-0002-8017-4005.
